# HTLV-1-Associated Myelopathy/Tropical Spastic Paraparesis Is Not Associated with SNP rs12979860 of the IL-28B Gene

**DOI:** 10.1155/2015/804167

**Published:** 2015-11-01

**Authors:** Antonio C. R. Vallinoto, Bárbara Brasil Santana, Keyla S. G. Sá, Tuane C. S. Ferreira, Rita Catarina M. Sousa, Vânia N. Azevedo, Rosimar N. M. Feitosa, Luiz Fernando A. Machado, Marluísa O. G. Ishak, Ricardo Ishak

**Affiliations:** ^1^Laboratório de Virologia, Instituto de Ciências Biológicas, Universidade Federal do Pará, Guamá, 66075-110 Belém, PA, Brazil; ^2^Núcleo de Medicina Tropical, Universidade Federal do Pará, Guamá, 66050-240 Belém, PA, Brazil

## Abstract

The present study investigated the association between the rs12979860 polymorphism in the IL-28B gene and HTLV-1 infection as well as the development of HTLV-1-associated myelopathy/tropical spastic paraparesis (HAM/TSP). HTLV-1-infected patients (26 HAM/TSP symptomatic and 53 asymptomatic) and 300 seronegative healthy controls were investigated. Plasma levels of the cytokines TNF-*α*, TNF-*β*, IL-8, IL-10, IL-6, and IFN-*γ* from infected patients were measured using an indirect enzyme-linked immunosorbent assay. The HTLV proviral load was measured using a real-time PCR assay, and T-cell subset counts were determined by flow cytometry. Real-time PCR was used to genotype the rs12979860 SNP. The allelic and genotypic distributions displayed no significant differences among the investigated groups. No significant association between the serum cytokine levels and the presence of the rs12979860 SNP in symptomatic and asymptomatic subjects was observed. A positive correlation (*p* = 0.0015) between TNF-*β* and IFN-*γ* was observed in the asymptomatic group, but a positive correlation was only observed (*p* = 0.0180) between TNF-*α* and IL-6 in the HAM/TSP group. The proviral load was significantly higher in HAM/TSP patients than in asymptomatic subjects. The present results do not support a previous report indicating an association between the SNP rs12979860 and HAM/TSP outcome.

## 1. Introduction

Human T-cell lymphotropic virus 1 (HTLV-1) is a member of the Retroviridae family and* Deltaretrovirus* genus [1]. HTLV-1 infection occurs worldwide and affects approximately 15 to 20 million individuals on all continents [2–4].

Most individuals infected with HTLV-1 remain asymptomatic for life, but 5–10% develop clinical symptoms [5, 6]. HTLV-1-associated myelopathy/tropical spastic paraparesis (HAM/TSP) and adult T-cell leukemia/lymphoma (ATLL) are the primary clinical manifestations affecting HTLV-1-infected subjects [1, 7].

Although recent studies indicate the possible use of genetic biomarkers as predictive factors associated with the outcome of the clinical manifestations [8–10], the involvement of these markers had remained unknown until now. Furthermore, HAM/TSP pathogenesis involves an immune-mediated response characterized by inflammatory mechanisms, which lead to the chronic destruction of myelin in the central nervous system [7, 11].

Interleukin-28B (IL-28B) is a member of a recently characterized family of cytokines known as interferon *λ* (IFN-*λ*1, IFN-*λ*2, and IFN-*λ*3 or interleukin-29, interleukin-28A, and interleukin-28B, resp.) [12]. IFN-*λ* is induced by the same stimulus that induces IFN-*α*/*β* [13, 14], and studies have revealed that IFN-*λ* inhibits viral replication [15, 16].

During a viral infection, Toll-like receptor-3, Toll-like receptor-4, and Toll-like receptor-7 recognize dsRNA and ssRNA in the cytoplasm or endosomes, activating RIG-1, NF*κ*B, IRF3, and IRF7 which stimulate the expression of the* IL-28B* gene [17–21]. When secreted, IL-28B binds their receptors and activates JAK-STAT-1/2 signaling pathways promoting transcription of interferon-stimulated genes (ISGs) that can result in expression of protein kinase RNA-activated (PKR), O-acetyl-L-serine (OAS), and proinflammatory cytokines [12, 22, 23]. Recently, Treviño et al. [24] reported an association between IL-28B polymorphisms and HAM/TSP outcome in Spanish patients, but those results were contested by Sanabani et al. [25] in a southeastern Brazilian population.

Regarding these controversial results, the present study investigated whether the rs12979860 polymorphism in the IL-28B gene is associated with HTLV-1 infection and the progression to HAM/TSP in patients from the Amazon region of Brazil, a population ethnically different from the previously studied Spanish and southeastern Brazilian populations.

## 2. Material and Methods

### 2.1. Populations Studied

The study group included 79 HTLV-1-infected subjects (26 HAM/TSP symptomatic and 53 asymptomatic) attending the outpatient clinic of the Tropical Medicine Nucleus of the Federal University of Pará and 300 seronegative healthy controls from the Virus Laboratory. To avoid confounding factors associated with ethnic origin, which eventually could cause sample bias and influence the genotypic and allelic frequencies, both groups were composed of subjects residing in Belém and had the same ethnic origin. All individuals were recruited between May 2005 and January 2012 and signed an informed consent form.

### 2.2. Obtaining Samples

Blood samples were collected in Vacutainer tubes containing K3-EDTA (Becton & Dickinson, Cambridge, UK) as an anticoagulant to obtain plasma and peripheral blood mononuclear cells (PBMCs). The samples were directed to the Virus Laboratory of the Biological Sciences Institute of the Federal University of Pará and stored at −20°C before use. The samples were previously screened for anti-HTLV using an enzyme-linked immunosorbent assay (Ortho Diagnostic Systems Inc., USA), and infection was confirmed by nested PCR as previously described [26]. The clinical diagnosis of HAM/TSP followed the criteria previously described [27].

### 2.3. Cytokine Levels

The plasma levels of cytokines TNF-*α*, TNF-*β*, IL-8, IL-10, IL-6, and IFN-*γ* from patients were evaluated by indirect enzyme-linked immunosorbent assays (Human ELISA Ready-SET-Go, EBioscience, Inc., San Diego, CA).

### 2.4. LTCD4^+^/LTCD8^+^ Count and Proviral Load Levels

The HTLV-1 proviral load and CD4^+^ T lymphocyte counts in HTLV-1-infected individuals were assessed at the time of entry into the study. Blood samples were processed within 4 hours of collection, and T-cell subset counts were determined by flow cytometry (FACScount, Becton & Dickinson, USA) using the FACScount reagent immunomonitoring kit according to a standard protocol recommended by the manufacturer (Becton & Dickinson, USA). The quantification of the HTLV proviral load was performed using a real-time PCR assay as previously described [28].

### 2.5. IL-28B Polymorphism

Real-time PCR (qPCR) was used to genotype the single nucleotide polymorphism (SNP) rs12979860 using the TaqMan Gene Expression Assay AHCS19G kit (Applied Biosystems, Foster City, CA, USA) following the technical procedures recommended by the manufacturer. A custom-designed assay provided by Applied Biosystems was used. The following primer sequences were used in these assays: forward primer, 5′-GCCTGTCGTGTACTGAACCA-3′; reverse primer, 5′-GCGCGGAGTGCAATTCAAC-3′; and the probes (VIC)-5′-TGGTTCGCGCCTTC-3′ and (FAM)-5′-CTGGTTCACGCCTTC-3′ for the C and T alleles, respectively.

The PCR reactions were prepared using TaqMan Universal PCR Master Mix components (Applied Biosystems, Foster City, CA, USA), which consisted of nucleotides, buffer, UNG, AmpliTaq, and a passive reference dye (ROX). The reaction mixture contained 5.0 *μ*L of Master Mix, 3.5 *μ*L of H_2_O, 0.5 *μ*L of IL-28B assay components (primer set and probe), and 1.0 *μ*L of DNA from each sample. The final volume for each reaction was 10 *μ*L.

The Step One Plus Real-Time PCR System (Applied Biosystems, Foster City, CA, USA) was used to perform the qPCR experiments with the following cycling protocol: one cycle of 60°C for 2 minutes, one cycle of 95°C for 10 minutes, and 50 cycles of 95°C for 15 seconds and 60°C for 20 seconds.

The program StepOne v2.2 (Applied Biosystems, Foster City, CA, USA) was employed to interpret the reaction results using the graphical representation of VIC and FAM fluorophore emissions with respect to constitutive ROX emissions.

### 2.6. Statistical Analysis

The genotypic and allelic frequencies observed were obtained by direct counting, and comparisons between frequencies were calculated using the Chi-square test. The association analysis between the genotype frequencies and the arithmetic average of the values of proviral load, cytokines, and TCD4^+^ and TCD8^+^ lymphocyte counts were performed using the Mann-Whitney test. Pearson's linear correlation analysis was used to compare the cytokine levels. Hardy-Weinberg equilibrium was calculated using the software BioEstat 5.0 [29]. A *p* value < 0.05 was considered statistically significant.

## 3. Results

### 3.1. Allelic and Genotypic Frequencies

The allele and genotype distributions displayed no significant differences between HTLV-infected subjects and healthy controls as well as between HAM/TSP and asymptomatic patients (Table 1). The CT genotype was the most prevalent among the groups analyzed, ranging from 44.3 to 50%, and the same predominance was observed for allele C, which displayed frequencies ranging from 51.67 to 52.83%. Hardy-Weinberg equilibrium was observed in both groups. Furthermore, the odds ratio analysis demonstrated that the SNP rs12979860 was not related to susceptibility or resistance to HTLV-1 infection or to the clinical prognosis of the infection.

### 3.2. Cytokine Levels

**Figure 1:**
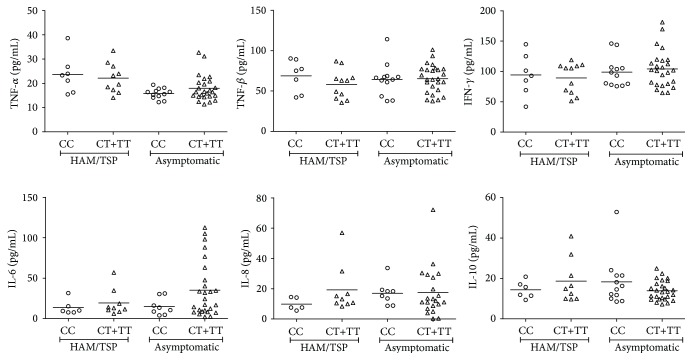
Cytokine serum levels according to the presence of the SNP rs12979860 in the HAM/TSP and asymptomatic groups.

**Figure 2:**
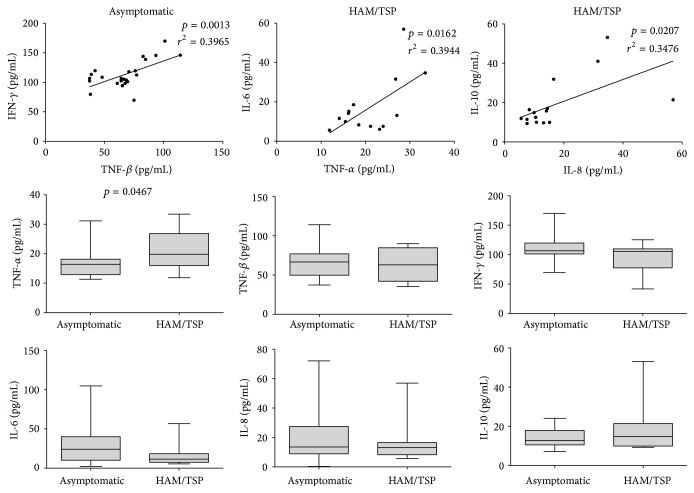
Comparison and correlation of the cytokine serum levels in the HAM/TSP and asymptomatic groups.

No significant association (Figure 1) was observed between cytokine levels and the presence of rs12979860 (genotypes CT+TT). However, when the cytokine levels were measured according to the presence or absence of HAM/TSP symptoms (Figure 2), a significantly increased level of TNF-*α* was observed in the HAM/TSP patients. In an attempt to correlate each cytokine value with each other (Figure 2), a positive correlation (*p* = 0.0013; *r* ^2^ = 0.3965) between TNF-*β* and IFN-*γ* was observed in the asymptomatic group, whereas TNF-*α* and IL-6 (*p* = 0.0162; *r* ^2^ = 0.3944) as well as IL-10 and IL-8 (*p* = 0.0207; *r* ^2^ = 0.3476) displayed positive correlations in the HAM/TSP group.

### 3.3. Proviral Load and CD4^+^/CD8^+^ T-Cells Levels

**Figure 3:**
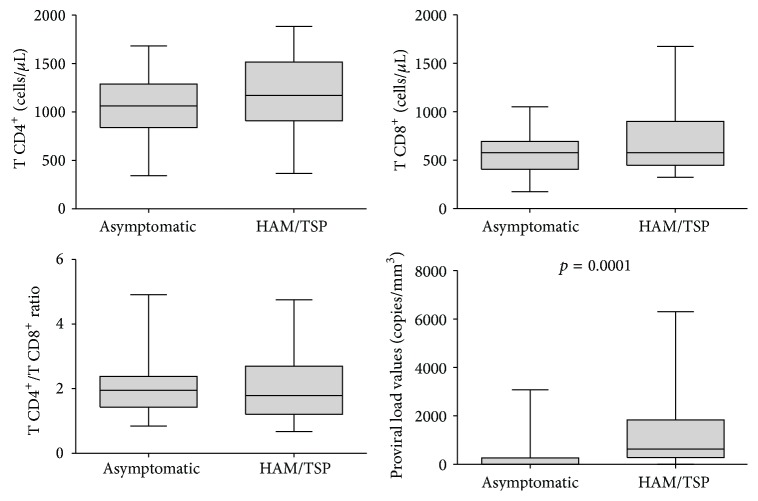
CD4^+^/CD8^+^ T-cell and proviral load measurements in the HAM/TSP and asymptomatic groups.

**Figure 4:**
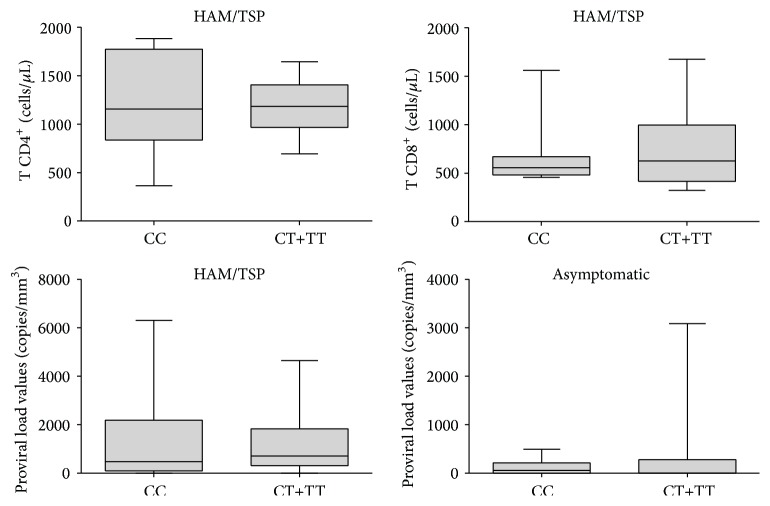
CD4^+^/CD8^+^ T-cell and proviral load measurements in the HAM/TSP and asymptomatic groups according to the presence of the SNP rs12979860.

The proviral load was significantly increased in HAM/TSP patients compared with asymptomatic subjects (*p* = 0.0002; Figure 3), but no association with the presence of the rs12979860 polymorphism was observed (Figure 4). No association was observed between the CD4^+^ and CD8^+^ T lymphocyte levels and symptomatic and asymptomatic infected subjects or the presence or absence of the SNP rs12979860.

## 4. Discussion

Symptomatic HTLV infection has been described in 5–10% of infected individuals [5], who may manifest mainly ATLL and HAM/TSP as the two most serious diseases associated with HTLV infection [1, 7, 30]. Several studies have attempted to associate the presence of SNPs in the host's genome with susceptibility to infection or the outcome of clinical manifestations in infected subjects [8–10, 31]. Recently, Treviño et al. [24] investigated the prevalence of the rs12979860 polymorphism in the IL-28B gene in a Spanish group and reported an increased frequency of the CT/TT genotypes in HAM/TSP patients compared with asymptomatic subjects. Furthermore, these authors reported a greater median proviral load in carriers of the CT/TT genotypes, suggesting the influence of this genetic background as an important biomarker for follow-up in the HTLV-infected individuals.

The results presented herein do not agree with this hypothesis given that we did not observe any differences in the allelic and genotypic frequencies among the symptomatic patients, asymptomatic patients, and control individuals. Furthermore, according to our results, the studied SNP had no influence on the HTLV proviral load, confirming the data previously obtained in a southern Brazilian population [25]; thus, our results do not support the use of this SNP as a predictive factor in the development of HAM/TSP. A possible explanation for the lack of agreement is the existence of distinct ethnic differences between the Brazilian and Spanish populations examined. The significantly increased levels of the HTLV-1 proviral load in HAM/TSP patients compared with asymptomatic subjects are a typical finding that has been commonly used as a biomarker for HAM/TSP prognosis [32].

The pathogenesis of HAM/TSP is based on the host's immunological response against infected cells, and cytokines play a central role in the regulation of the CD8^+^ T lymphocyte response against HTLV-1 to control virus replication and HTLV-infected cell proliferation [6, 33]. According to our results, no association was observed between the IL-28B genotypes and cytokine plasma levels; however, significantly increased serum levels of TNF-*α* were observed in the HAM/TSP group compared with the controls. Furthermore, a positive correlation between TNF-*β* and IFN-*γ* was evident in the asymptomatic group. These results may reflect the systemic antiviral cellular response against CD4^+^ T cells infected with HTLV-1. Positive correlations between TNF-*α* and IL-6 as well as IL-10 and IL-8 were observed in the HAM/TSP group, and these results are a potential consequence of the proinflammatory immune responses typically observed in this group of patients. Lal and Rudolph [34] reported increased IL-6 and TNF-*α* levels in culture supernatants from HTLV-1/HTLV-2-infected individuals compared with normal controls, suggesting that these cytokines may regulate lymphocyte proliferation in infected individuals. The* in vivo* results of Starling et al. [32] demonstrate increased serum concentrations of IL-6 and TNF-*α* in HAM/TSP patients compared with asymptomatic subjects.

## 5. Conclusion

Knowledge regarding the immunogenetic and virological profile of HTLV-infected subjects can guide patient clinical counseling, but our results did not reveal any association between the SNP rs12979860 and the TSP/HAM outcome, refuting previous evidence. However, population-scale genetic studies involving larger samples and different ethnic groups are recommended to confirm these results.

## Figures and Tables

**Table 1:** Genotypic and allelic frequencies of the SNP rs12979860 in HTLV-1-infected patients and healthy control subjects.

SNP rs12979860 (C>T)	HTLV patients (*n* = 79) *n* (%)	Control group (*n* = 300) *n* (%)	OR	*p*	TSP/HAM (*n* = 26) *n* (%)	Asymptomatic (*n* = 53) *n* (%)	OR	*p*
Genotype								
CC	24 (30.38)	80 (26.67)	1.2000	0.6056	09 (34.61)	15 (28.30)	1.3412	0.7543
CT	35 (44.30)	150 (50.00)	0.7955	0.4385	09 (34.61)	26 (49.06)	0.5498	0.3305
TT	20 (25.32)	70 (23.33)	1.1138	0.8259	08 (30.77)	12 (22.64)	1.5185	0.6133
Allele								
C	83 (52.53)	310 (51.67)	1.0353	0.9171	27 (51.92)	56 (52.83)	0.9643	0.9504
T	75 (47.47)	290 (48.33)			25 (48.08)	50 (47.17)		

OR: odds ratio.
